# Early Changes of VEGF Levels After Zoledronic Acid in Women With Postmenopausal Osteoporosis: A Potential Role of Vitamin D

**DOI:** 10.3389/fmed.2021.748438

**Published:** 2021-11-18

**Authors:** Federica Bellone, Antonino Catalano, Angelo Ruggero Sottile, Agostino Gaudio, Saverio Loddo, Francesco Corica, Nunziata Morabito

**Affiliations:** ^1^Department of Clinical and Experimental Medicine, University of Messina, Messina, Italy; ^2^Department of Clinical and Experimental Medicine, University of Catania, Catania, Italy

**Keywords:** bisphosphonate, zoledronic acid, vascular endothelial growth factor, osteonecrosis, postmenopausal, osteoporosis, vitamin D

## Abstract

Zoledronic acid (Zol) is a widely used intravenous aminobisphosphonate to treat both benign and malignant skeletal diseases, and bisphosphonate-related osteonecrosis of the jaw (BRONJ) is a serious side effect whose pathophysiology remains poorly understood. Vascular Endothelial Growth Factor (VEGF) has been recognized to mediate BRONJ in cancer patients undergoing Zol treatment, however data on VEGF are lacking in patients with osteoporosis. Increasing evidences demonstrate that vitamin D influences VEGF levels. The aim of this study was to investigate the influence of Zol on VEGF levels and the possible role for vitamin D on the Zol mediated changes of VEGF concentration in women with postmenopausal osteoporosis. Twenty-eight postmenopausal women with osteoporosis were enrolled and randomized into two groups to receive Zol (5 mg) or placebo. At baseline, at day-3 and day-30 VEGF serum levels were measured; bone turnover markers, 25-hydroxyvitamin D [25(OH)D] and serum calcium were evaluated at baseline. In Zol-treated women, VEGF increased significantly on day-3, and then decreased on day-30. In the Zol-treated women, the percent change of VEGF levels between baseline and day-30 (−18% at day-30 vs. baseline, *p* = 0.01) was significantly associated with serum 25(OH)D values (*r* = 0.29, *p* = 0.028). At a stepwise multiple regression analysis, after correcting for age, BMI, time since menopause, femoral neck BMD, osteocalcin, C-terminal telopeptide of type 1 collagen, and baseline VEGF levels, 25(OH)D levels were independently associated with VEGF change (β = 1.7, SE = 0.71, *p* = 0.03). For the first time, we detected early modifications of circulating VEGF in postmenopausal women receiving Zol for osteoporosis, identifying a vitamin D-dependent modulation of these changes.

## Introduction

Zoledronic acid (Zol) is a powerful intravenous aminobisphosphonate (N-BP) currently used against primary or secondary osteoporosis (1). As shown in a randomized, double-blind, placebo-controlled, multinational study, the HORIZON trial, when compared with the placebo control group, Zol 5 mg administered once a year for up to 3 years was demonstrated to increase bone mineral density (BMD) at different skeletal regions, improve bone metabolism markers and reduce the risk of new morphometric vertebral and hip fractures (2, 3). Zol treatment is commonly safe and well-tolerated, with the most frequent adverse events being transient and reversible (4).

Osteonecrosis of the jaw (ONJ) represents an emergent severe oral disease, with dramatic implications for health related patient's quality of life (5). Since 2003, there has been a surge of interest in the effects of BPs on oral health, and ONJ has been described as a BP related class effect, and the acronym “BRONJ” was introduced to indicate Bisphosphonate-Related Osteonecrosis of Jaw. The American Association of Oral and Maxillofacial Surgeons (AAOMS) defines BRONJ as an exposure of portions of the jawbone in patients who have been exposed to BPs, lasting more than 8 weeks in patients with no history of jaw irradiation (6). Nonetheless, recent studies have clearly shown that also the antiresorptive agent denosumab and some antiangiogenic drugs could similarly induce ONJ (7, 8). Accordingly, the term Medication Related Osteonecrosis of Jaw (MRONJ) has been also acknowledged in medical literature (9, 10). In metastatic cancer patients, the risk of developing BRONJ has been mainly associated with BP potency, high, and cumulative dose as well as intravenous administration (11, 12). BRONJ has been rarely reported in osteoporotic postmenopausal women (13).

In accordance with published data, the angiogenesis suppression may play a role in the pathophysiology of BRONJ, which is still uncertain and under debate (14, 15). Beyond the known antiresorptive, immunomodulating, and direct antitumor actions, BPs are actually considered also antiangiogenic drugs. Particularly, Zol has been proven to decrease VEGF concentrations in patients with metastatic bone cancer (16, 17), and the decrease of serum VEGF levels after intravenous BPs has been proposed as a possible early predictive marker of BRONJ in cancer patients (18).

Postmenopausal women exhibit a higher risk of periodontal diseases due to the fall of estrogen levels, which exert a trophic action on oral cavity (19). Scardina et al. showed a decreased periodontal capillary density, an increased tortuosity and decreased diameter of loops in oral microcirculation in a setting of postmenopausal women (20). Consequently, the oral microcirculatory alterations observed in postmenopause may encourage the adverse effects of some medications, such as Zol, on oral health.

Moreover, conflicting evidences demonstrated that vitamin D can influence VEGF levels: some data support a promoting action, others a suppressive one of vitamin D upon the release of VEGF (21–23). An adequate vitamin D status has been claimed to amplify and maintain over time the effect of BPs, even after their discontinuation (24), and the risk to develop BRONJ has been shown higher in BPs treated patients with concomitant hypovitaminosis (25).

Therefore, it remains still unclear whether a therapeutic dose of Zol for the medical management of osteoporosis in postmenopausal women have anti-angiogenic effects, due to post-dose changes of VEGF, as previously recognized in cancer patients.

The aim of this study was to explore the *in vivo* effect of Zol on circulating VEGF levels and the possible role for vitamin D on the Zol mediated VEGF perturbancies in a cohort of postmenopausal women treated for osteoporosis.

## Materials and Methods

### Study Subjects

This prospective randomized placebo-controlled study considered a cohort of Caucasian postmenopausal women attending the Center for Prevention, Diagnosis and Treatment of Osteoporosis in the Department of Internal Medicine of the University of Messina. Participants were consecutively recruited if affected by at least one prevalent vertebral fracture and with a BMD T-score values indicative of osteopenia or osteoporosis (in accordance with the World Health Organization diagnostic criteria), which were eligible for i.v. therapy with Zol, in accordance with standards of good clinical practice. Exclusion criteria were moderate to severe chronic kidney or liver failure, secondary causes of osteoporosis including hyperthyroidism, hyperparathyroidism, hypercortisolism, or malabsorption, *in situ* or systemic disease that could have impaired microcirculation such as diabetes mellitus, dyslipidemia, arterial hypertension, connective tissue diseases, autoimmune disorders or oral lichen planus. Patients undergoing treatment for osteoporosis (e.g., BPs, teriparatide, denosumab, selective estrogen receptor modulators, strontium ranelate, and calcitonin) or with a history of prior therapy with these bone agents were also not considered.

Participants were enrolled after a careful clinical check including dental examination with preventive dentistry; women with poor oral hygiene, periodontal diseases, poorly fitting dentures, history of dental diseases, invasive dental procedures, e.g., tooth extractions, or with unhealed open soft tissue lesions in the mouth were not considered for this study.

Fracture risk was assessed by using Fracture Risk Assessment Tool (FRAX), a computer-based algorithm (http://www.shef.ac.uk/FRAX) that estimates the 10-year probability of a major fracture (hip, clinical spine, humerus, or wrist fracture) and the 10-year probability of a hip fracture, as previously reported (26). All the patients were under vitamin D supplementation (cholecalciferol 25,000 IU every 2 weeks) and they took calcium (calcium carbonate 500–1,000 mg daily) when needed to reach the recommended daily calcium intake.

Recruited women were randomized into two groups using a computer-generated randomization schedule in the order in which they were enrolled in the study.

At baseline, 18 participants underwent a single Zol 5 mg/100 mL (Aclasta®) administration at 10.00 a.m. and within 30 min. Ten participants, not receiving Zol served as controls and received i.v. 100 mL saline solution 0.9%.

The study was approved by our institutional research committee Policlinico “G. Martino” Messina (Prot.n. 71/19); it was performed in accordance with the 1964 Declaration of Helsinki and its later amendments for experiments involving humans and uniform requirements for manuscripts submitted to biomedical journals. Signed informed consent was obtained from all the participants.

### Biochemical Assays

Serum samples were collected just before Zol administration at baseline, and then at day-3 and day-30 after Zol administration. Serum was separated from the blood corpuscles by centrifugation and stored frozen at −80°C until analyzed.

VEGF-A serum levels were measured at each time-point by Enzyme-Linked Immunosorbent Assay, ELISA (Duoset, R&D Systems Europe Ltd., Abingdon, UK) according to the manufacturer's instructions. The limit of detection of human VEGF-A defined as the analyte concentration resulting in an absorption significantly higher than that of the dilution medium (mean plus two standard deviations) was determined to be 7.9 pg/mL (mean of 6 independent assays).

The inter- and intra-assay coefficient of variation was 4.3 and 6.2%, respectively.

At baseline, levels of Osteocalcin (BGP), Bone-specific alkaline phosphatase (BSAP) as markers of bone formation, and serum C-telopeptide of type 1 collagen (CTX), as marker of bone resorption, 25-hydroxyvitamin D (25(OH)D) and serum calcium were recorded.

BGP was measured by immunoenzymatic assay (Invitrogen Ltd., UK) with an intra-assay coefficient of variance (CV) of 3.1% and inter-assay CV of 3.5%; BSAP was measured by immunoenzymatic assay with the Access Immunoassay System (Beckman Coulter, Fullerton, California) with intra- and interassay CVs of 2.3–3.7% and 4.9–9.8%, respectively; CTX was assessed using the Elecsys 2010 Immunoassay System (Roche, Basel, Switzerland) with intraassay CVs of 1.6% to 3% and interassay CVs of 1.3–4.3%; levels of 25(OH)D were detected by high performance liquid chromatography; serum calcium was measured using standard laboratory techniques.

### Bone Mineral Density and Vertebral Fractures

At baseline, a Dual-energy X-ray Absorptiometry (DXA) densitometer (Hologic Discovery) was used to assess BMD at the lumbar spine (L1–L4) and at the femoral neck. The DXA calibration was performed daily following the manufacturer's instruction. Its coefficient of variation (CV) was 0.5%. Vertebral fractures (Vfs) were detected by a conventional spinal radiograph and images were taken from anterior-posterior (AP) and lateral views applied of the thoracic and lumbar spine.

Vfs was defined in accordance to the semiquantitative method proposed by Genant: a fracture was diagnosed if a 20% reduction in the anterior or middle height compared to the posterior height of the same vertebra or the posterior height compared to the superior and anterior adjacent ones was detected (27).

### Statistical Analysis

Statistical analyses were performed using MedCalc software (version 10.2.0.0; MedCalc Software, Mariakerke, 173 Belgium). Values were expressed as mean ± SD or median (IQR). The normal distribution of values was verified with the Kolmogorov-Smirnov test. Differences were evaluated using the Student's *t*-test for paired and unpaired observations, with Wilcoxon test and Mann-Whitney test, and repeated measures analysis of variances. Pearson's correlation coefficient was calculated to evaluate the degree of association between two variables. A multiple regression analysis was performed to determine the influence of one independent variable after correcting for the others. A *post-hoc* analysis was performed to explore change of VEGF levels in participants who experienced or not an Acute Phase Response (APR) after Zol administration. For all the tests, a *P*-value of 0.05 or less was considered to indicate statistical significance.

## Results

Baseline clinical features and main laboratory data of the participants are shown in Table 1. Particularly, at baseline, no significant differences in any of the investigated variables were observed between both groups of women.

**Table 1:** Main clinical features of recruited women at baseline.

	**Zol (*n* = 18)**	**Controls (*n* = 10)**	***p***
Age (yr)	62.5 (56.3–73.8)	61.3 (55.4–70.9)	0.73
Age at menopause (yrs)	45.3 ± 5.4	48.6 ± 4.1	0.12
Time since menopause (yr)	18.2 ± 7.4	15.3 ± 6.5	0.31
BMI (Kg/m^2^)	23.0 (21.2–25.1)	23.2 (21.6–26.2)	0.79
Current smoking [n(%)]	2 (10)	1 (10)	NS
Alcohol ≥ 3units/day [n(%)]	0 (0)	0 (0)	NS
25(OH)D (ng/ml)	44.8 (40.6–53.1)	42.7 (32.1–49.2)	0.78
Osteocalcin (ng/mL)	7.0 (6.3–14.7)	7.8 (5.4–13.6)	0.89
CTX (ng/mL)	0.9 (0.2–1.1)	0.76 (0.3–0.98)	0.81
VEGF (pg/mL)	121.81 ± 86.36	106.72 ± 67.92	0.47
**Bone mineral density**			
Lumbar spine T-score (SD)	−2.62 ± 1.3	−2.71 ± 1.08	0.81
Lumbar spine BMD (gr/cm^2^)	0.75 ± 0.15	0.74 ± 0.18	0.79
Femoral neck T-score (SD)	−2.45 ± 0.35	−2.32 ± 0.51	0.43
Femoral neck BMD (gr/cm^2^)	0.58 ± 0.08	0.61 ± 0.1	0.39
**FRAX score**			
Major osteoporotic fracture (%)	28 ± 17	25 ± 14	0.63
Hip fracture (%)	12 ± 9	10 ± 8	0.56

*Data are expressed as mean ± SD, median (IQR) or number (%) as appropriate*.

*NS, not significant; Zol, Zoledronic acid; BMI, Body Mass Index; 25(OH)D,25-hydroxyvitamin D; CTX, C-telopeptide of type 1 collagen; VEGF, Vascular Endothelial Growth Factor; BMD, Bone Mineral Density; FRAX, Fracture Risk Assessment Tool*.

**Figure 1:**
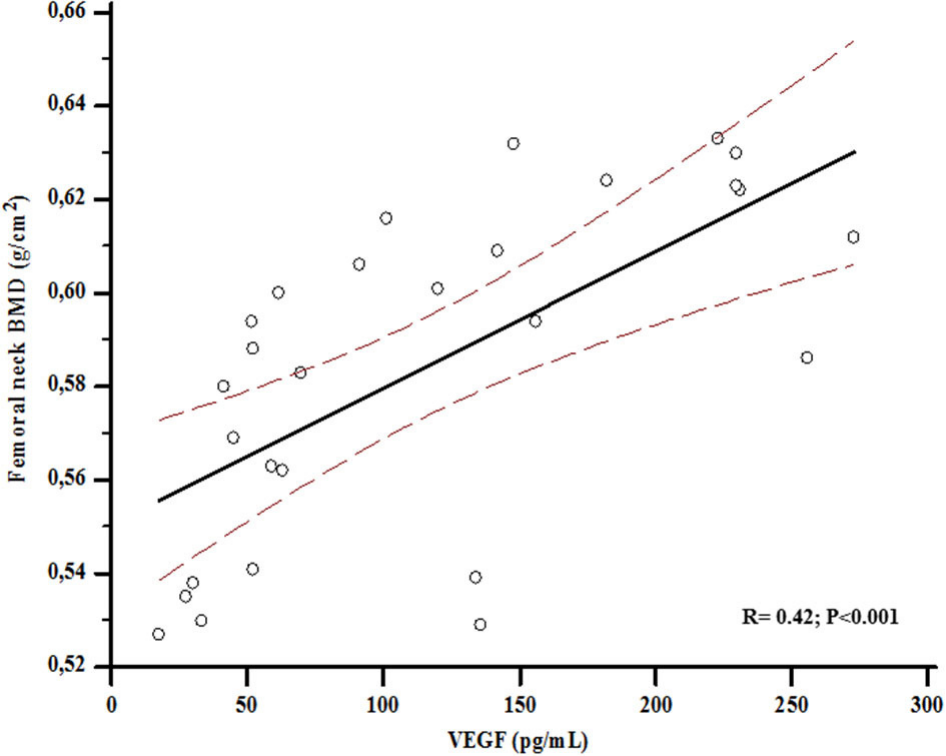
Association between bone mineral density (BMD) at femoral neck and Vascular Endothelial Growth Factor (VEGF) levels at baseline.

In the overall population (*n* = 28), serum VEGF values were positively associated with femoral BMD (*r* = 0.42; *p* < 0.001) (Figure 1), and this correlation was maintained even after adjusting for age and BMI (*r* = 0.58; *p* = 0.01).

**Figure 2:**
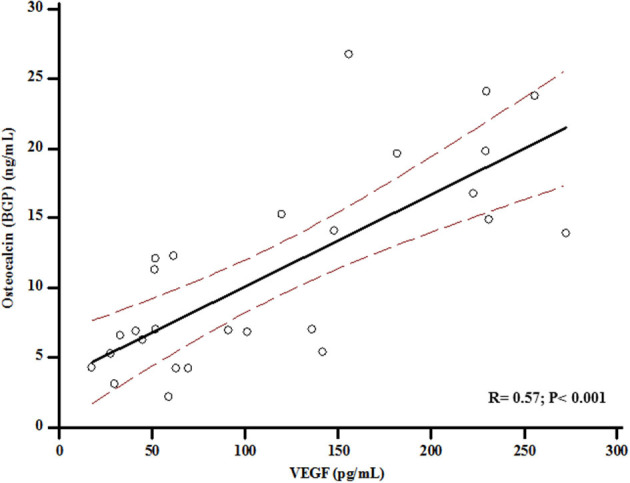
Association between levels of Osteocalcin and Vascular Endothelial Growth Factor (VEGF) at baseline.

**Figure 3:**
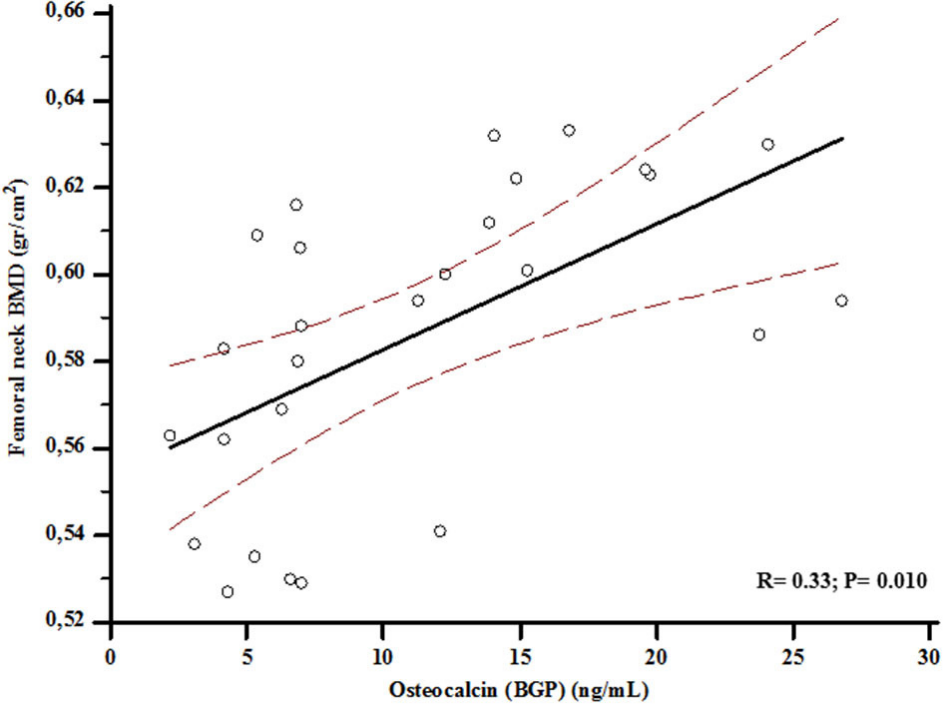
Association between levels of osteocalcin (BGP) and bone mineral density (BMD) at femoral neck at baseline.

At baseline, VEGF values were associated with BGP levels (*r* = 0.57; *p* < 0.001) (Figure 2), and serum BGP positively correlated with femoral neck BMD (*r* = 0.33; *p* = 0.003) (Figure 3).

**Figure 4:**
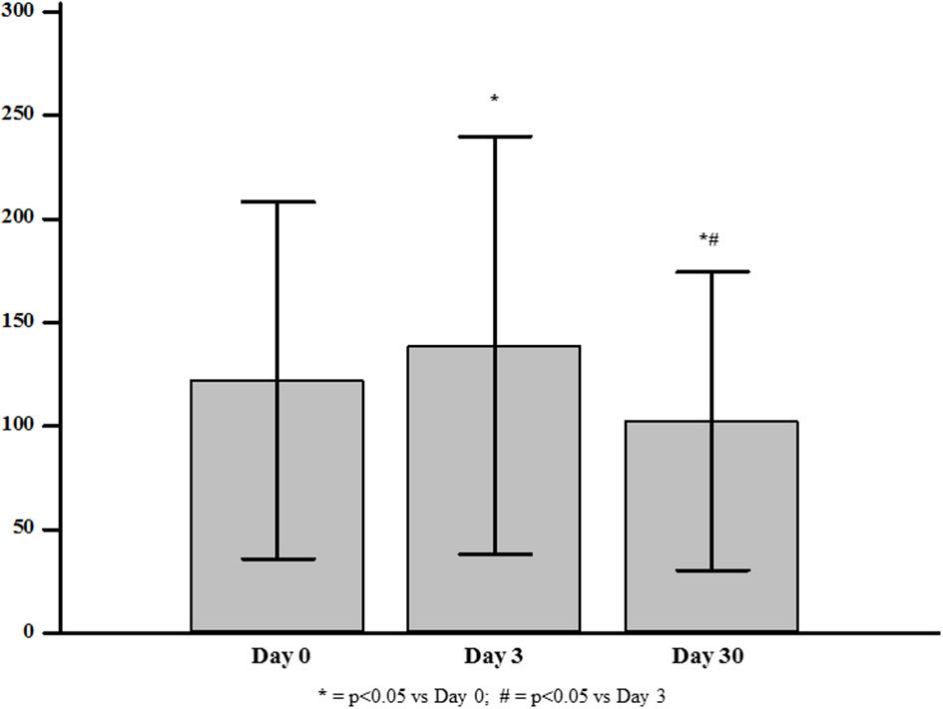
Levels of Vascular Endothelial Growth Factor (VEGF) at baseline and at day-3 and day-30 after zoledronic acid administration.

**Figure 5:**
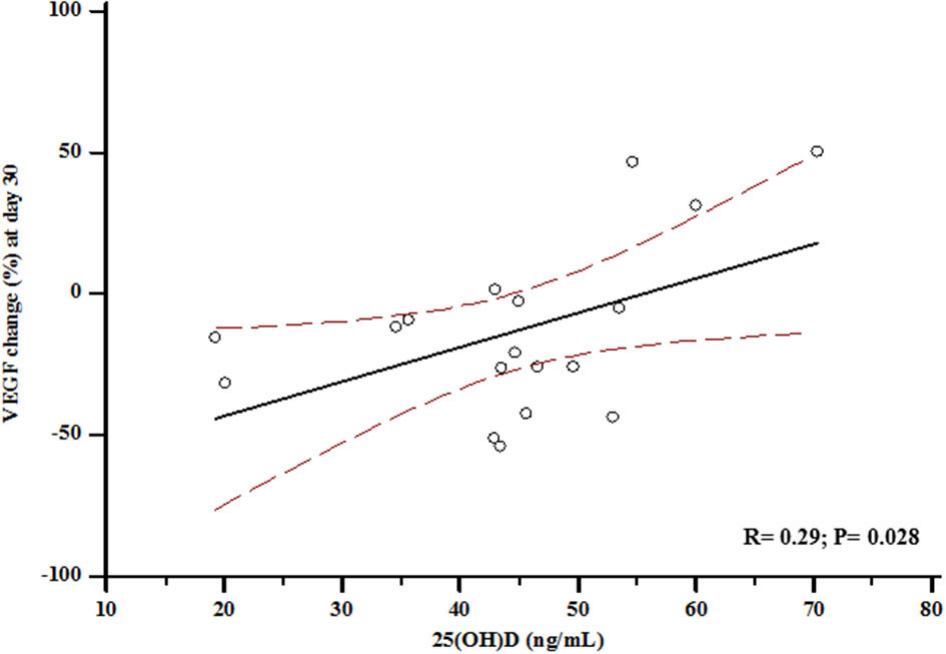
Association between vitamin D status at baseline and percent change of Vascular Endothelial Growth Factor (VEGF) at day-30.

In the 18 participants who received Zol, median VEGF levels significantly increased at day-3 to a value of 138.83 ± 100.89 pg/mL (+13% vs. baseline, *p* = 0.04), and then decreased at day-30 to a value of 102.43 ± 72.21 pg/mL (−26% vs. day-3, *p* = 0.002 and −18% vs. baseline, *p* = 0.01) (Figure 4). Conversely, controls did not show any significant changes over time (114.61 ± 68.13 and 110.72 ± 71.12 pg/mL at day-3 and day-30, respectively; *p*_all_ > 0.5 vs. baseline). In the Zol-treated women, the percent change of VEGF levels between baseline and day-30 was significantly associated with serum 25(OH)D values (*r* = 0.29, *p* = 0.028) (Figure 5). Change of VEGF at day-3 was also associated with CTX levels (*r* = 0.77, *p* = 0.003). At a stepwise multiple regression analysis, 25(OH)D levels were the only predictor of VEGF change, after correcting for age, BMI, time since menopause, femoral neck BMD, BGP, CTX and baseline levels of VEGF (β = 1.7, SE = 0.71, *p* = 0.03). A *post-hoc* analysis showed that the rise of VEGF at day-3 was higher in the 3 participants who suffered APR in comparison with the other women receiving Zol who did not suffer APR [153.85 ± 126.5 vs. 135.15 ± 100.1 (+13%, *p* = 0.3)], although not reaching a statistical significance.

## Discussion

Zol is the most potent and long-acting N-BP used for the treatment of osteoporosis, Paget's disease, myeloma, and cancers to reduce adverse skeletal related events (1). Change of VEGF levels have been previously reported in cancer patients receiving Zol (17), and decreased VEGF values have been also associated with the BRONJ risk (18).

To the best of our knowledge, this is the first prospective cohort study investigating the early serum changes of VEGF levels in high fracture risk postmenopausal women receiving Zol. Particularly, VEGF levels significantly raised soon after 3 days from Zol administration, and then decreased after 30 days to a value below the baseline one.

The raise of VEGF at day-3 may fall in the window of the frequent occurring APR after i.v. N-BP administration.

APR is a common side effect observed in 10 to 50% of patients after the first Zol dose. It consists of a systemic immune reaction induced by the stimulation of circulating γδ T cells and the subsequent release of several inflammatory cytokines as interleukin 6 (IL-6), tumor necrosis factor-β (TNF-β) and interferon-γ (IFN-γ) (28, 29). Related symptoms occur within 24–36 h and usually disappear in about 3 days (30).

VEGF is a specific endothelial cell mitogen and a strong vascular permeability factor; thus, it may be considered as a proinflammatory cytokine. Consequently, in patients receiving Zol, the early increase of VEGF levels could be a biochemical mark of VEGF involvement in the cytokine storm leading to APR (31). Accordingly, at a *post-hoc* analysis, the rise of VEGF at day-3 was 13% higher in the 3 participants who suffered APR in comparison with the other women receiving Zol who did not experienced APR, although without significant difference.

On the other hand, the decrease of VEGF levels after 30 days could be determined by the well-known Zol related suppression of bone turnover and Zol activity on endothelial cells. Osteoclasts, osteoblasts, osteocytes and endothelial cells are sources of VEGF (32). In the bone tissue, VEGF stimulates angiogenesis, osteoblast differentiation, and finally bone formation and bone healing. Furthermore, VEGF has been shown to promote osteoclast differentiation and survival (33). Accordingly, VEGF could orchestrate the crosstalk between endothelial, osteoblastic, and hematopoietic cells (34). Data on VEGF levels in osteoporotic patients are conflicting, and circulating VEGF could be influenced by treatment (35).

In all the participants of this study, at baseline, we observed a significant positive association between VEGF and both BGP and femoral BMD. These findings have not been previously described and add new insights about the possible VEGF key role in bone health; moreover, they are consistent with previous studies showing significantly lower VEGF levels in women with postmenopausal osteoporosis (36). Therefore, the significant association of VEGF with BGP we observed could suggest a potential role of VEGF as a marker of bone formation. The mechanical loading has been reported to enhance osteocyte production of various cytokines, including VEGF, regulating bone homeostasis: this could explain its association with BMD at femoral neck, showed in Figure 1 (37).

Several lines of evidence based on both *in vitro* and *in vivo* studies (17, 18, 38, 39) suggest the anti-angiogenic and anti-tumor VEGF-related Zol effect. However, few researches involved humans and mostly cancer patients. In 30 patients suffering from solid tumors with bone metastases, treated with a single infusion of Zol 4 mg, Santini et al. reported a significant reduction of VEGF levels after 2, 7, and 21 days, with the largest significance (*p* < 0.001) on day 21 (40). Moreover, low intermittent doses of Zol (1 mg every 7 days for 4 times followed by 4 mg every 28 days for 3 times), produced significant reductions of the circulating VEGF early after 7 days in cancer patients with bone metastasis from solid tumors. Moreover, after 84 days and a 16 mg cumulative Zol dose, VEGF levels tended to rise, although remaining significantly lower in comparison with baseline (40). Remarkably, even though the different dose and schedule, the reduction of serum VEGF levels observed in osteoporotic postmenopausal women in our study was of a smaller magnitude than that one seen in the oncology setting.

In women with postmenopausal osteoporosis, only one elegant study by Ishtiaq et al. evaluated the VEGF change *in vivo* and *in vitro* after N-BPs. Alendronate (Aln), administered at a weekly dose of 70 mg, produced a VEGF decline after 3 and 6 months, reaching a statistical significance after 12 months (*p* = 0.02). Consistently, Aln and Zol, in the culture medium of two osteoblastic cell lines, significantly reduced the VEGF production, confirming that N-BP related *in vivo* modification of this cytokine should be attributed to a direct effect on bone cells (21).

The VEGF reduction obtained *in vitro* with Zol was more pronounced than that one with Aln and it occurred at a lower dose, in accordance with higher Zol potency; surprisingly, the addition to the medium in osteoblastic cell lines of calcitriol, the active vitamin D metabolite, reverted the N-BP induced suppression of VEGF release (21).

An inadequate vitamin D status could even amplify the VEGF-mediated suppression of angiogenesis induced by N-BPs, and particularly by Zol; due to this pathophysiological mechanism, an increased prevalence of BRONJ in patients with vitamin D deficiency has been suggested by some researches (18, 25, 41, 42).

Our findings are consistent with *in vitro* data by Ishtiaq et al. (21), although the significance of the effect of N-BPs on the production of pro-angiogenic factors to *in vivo* pharmacology remain uncertain; our data showed that the serum 25(OH)D concentration, indicative of vitamin D status, was significantly associated with the VEGF change in postmenopausal women receiving Zol. In other terms, enrolled women, which already had normal vitamin D levels at baseline, showed a smaller reduction of VEGF serum levels at the end of the observation period the more the vitamin D levels were higher.

Among its extraskeletal effects (43–46), vitamin D has been proven to influence VEGF levels, although its relationship with this cytokine is still under debate and divergent data exist in the literature. Vitamin D has been suggested to enhance angiogenic factors in several *in vitro ed in vivo* studies (47–49). Particularly, vitamin D boosts the expression of VEGF in the cell culture of human umbilical vein endothelial cells (HUVECs) (44, 50, 51), and a direct overexpression of VEGF trough vitamin D receptor (VDR) activation by calcitriol has been reported in vascular smooth muscle cells (VSMCs) (44). In a recent study which used an animal model of pre-eclampsia, vitamin D deficiency lowered the placental protein levels of pro-angiogenic proteins VEGF; a low dose vitamin D supplementation beginning from pre-pregnancy and continuing through pregnancy normalized the levels of VEGF (47). On the other hand, a down-regulation of VEGF by 1,25(OH)2D and its metabolites has been reported *in vitro* (52), and consistently a vitamin D-induced suppression of VEGF levels has been observed in breast cancer women treated with tamoxifen (22) and in chronic spontaneous urticaria (23).

Emami et al. demonstrated also that vitamin D might decrease pro-angiogenic factors such as visfatin and in turn VEGF in ulcerative colitis patients with low 25(OH)D levels (53). Additionally, in breast cancer xenograft tumors, 1,25(OH)2D3 could induce apoptosis of VEGF sprouting endothelial cells, thereby reducing the blood vessel density modulating pathologic angiogenesis (54, 55).

The current knowledge about vitamin D and angiogenesis still remain not fully clarified and further focused researches are needed.

We must recognize this study has limitations including the small sample size, consisting of only women in postmenopausal age and the observation period not long enough to capture Zol-induced VEGF modifications in the long term. This study is not focused on BRONJ; thus, the observed changes of VEGF levels after Zol administration may have uncertain significance, and their pathophysiological role requires additional investigations. Thus, further studies, focused on osteoporotic postmenopausal women, with a large sample size and with a long follow-up, are needed to confirm our data and to address the association with BRONJ. Additionally, measuring other angiogenic factors could lead to a more in-depth profile of the effect of Zol on angiogenesis.

At the same time, our findings improve the knowledge of cytokine modifications after N-BP administration, suggesting a role for vitamin D on the Zol mediated VEGF perturbancies.

In conclusion, in osteoporotic postmenopausal women, Zol administration at a dose of 5 mg provoked early modifications of circulating VEGF and vitamin D modulated this VEGF change.

## Data Availability Statement

The datasets generated during and/or analyzed for this study are available from the corresponding author on reasonable request.

## Ethics Statement

The studies involving human participants were reviewed and approved by Institutional Research Committee Policlinico G. Martino Messina (Prot.n. 71/19). The patients/participants provided their written informed consent to participate in this study.

## Author Contributions

FB, AC, and NM designed the study. ARS, AC, and FB analyzed data and wrote the manuscript. AG, SL, and FC had full access to all the data in the study and take responsibility for the integrity and the accuracy of the data analysis. All authors reviewed the manuscript.

## Conflict of Interest

The authors declare that the research was conducted in the absence of any commercial or financial relationships that could be construed as a potential conflict of interest.

## Publisher's Note

All claims expressed in this article are solely those of the authors and do not necessarily represent those of their affiliated organizations, or those of the publisher, the editors and the reviewers. Any product that may be evaluated in this article, or claim that may be made by its manufacturer, is not guaranteed or endorsed by the publisher.
